# Trajectories of Dynamic Risk Factors as Predictors of Violence and Criminality in Patients Discharged From Mental Health Services: A Longitudinal Study Using Growth Mixture Modeling

**DOI:** 10.3389/fpsyt.2019.00301

**Published:** 2019-05-09

**Authors:** Mélissa Beaudoin, Stéphane Potvin, Laura Dellazizzo, Mimosa Luigi, Charles-Edouard Giguère, Alexandre Dumais

**Affiliations:** ^1^Centre de Recherche de l’Institut Universitaire en Santé Mentale de Montréal, Montreal, QC, Canada; ^2^Department of Psychiatry and Addictology, Faculty of Medicine, Université de Montréal, Montreal, QC, Canada; ^3^Institut national de psychiatrie légale Philippe-Pinel, Montreal, QC, Canada

**Keywords:** risk factors, violence, criminality, psychiatric patients, substance use, longitudinal study, growth mixture models

## Abstract

**Background:** Individuals with severe mental illnesses are at greater risk of offenses and violence, though the relationship remains unclear due to the interplay of static and dynamic risk factors. Static factors have generally been emphasized, leaving little room for temporal changes in risk. Hence, this longitudinal study aims to identify subgroups of psychiatric populations at risk of violence and criminality by taking into account the dynamic changes of symptomatology and substance use.

**Method:** A total of 825 patients from the MacArthur Violence Risk Assessment Study having completed five postdischarge follow-ups were analyzed. Individuals were classified into outcome trajectories (violence and criminality). Trajectories were computed for each substance (cannabis, alcohol, and cocaine, alone or combined) and for symptomatology and inputted as dynamic factors, along with other demographic and psychiatric static factors, into binary logistic regressions for predicting violence and criminality. Best predictors were then identified using backward elimination, and receiver operator characteristic (ROC) curves were calculated for both models.

**Results:** Two trajectories were found for violence (low versus high violence). Best predictors for belonging in the high-violence group were low verbal intelligence (baseline), higher psychopathy (baseline) and anger (mean) scores, persistent cannabis use (alone), and persistent moderate affective symptoms. The model’s area under the curve (AUC) was 0.773. Two trajectories were also chosen as being optimal for criminality. The final model to predict high criminality yielded an AUC of 0.788, retaining as predictors male sex, lower educational level, higher score of psychopathy (baseline), persistent polysubstance use (cannabis, cocaine, and alcohol), and persistent cannabis use (alone). Both models were moderately predictive of outcomes.

**Conclusion:** Static factors identified as predictors are consistent with previously published literature. Concerning dynamic factors, unexpectedly, cannabis alone was an independent co-occurring variable, as well as affective symptoms, in the violence model. For criminality, our results are novel, as there are very few studies on criminal behaviors in nonforensic psychiatric populations. In conclusion, these results emphasize the need to further study the predictors of crime, separately from violence and the impact of longitudinal patterns of specific substance use and high affective symptoms.

## Introduction

Violence is a complex and multifactorial issue that has serious health and social consequences (1). In comparison to the general population, both violence and criminality have been widely shown to be increased in those with psychiatric illnesses such as affective and psychotic disorders (2–5). The association between psychiatric illnesses and violence is complex mainly due to the interplay of a variety of static and dynamic factors (6, 7). It has been quite well established that risk factors for offending act in a cumulative and interactive manner (8). The state of knowledge suggests that the occurrence of violence is associated with a number of sociodemographic variables (e.g., age, gender, economic and social living status), substance use, a history of antisocial and violent acts, as well as psychopathic personality (9, 10). Yet, while there has been increasing literature on dynamic risk factors, research has paid more attention to risk status at a specific time point, which emphasizes static risk factors for violence (11).

To date, studies on patients with psychiatric disorders have not used models (i.e., growth mixture modeling) to predict violence and/or criminality with specific trajectories of dynamic risk factors. Longitudinal research using individual trajectory analyses may allow for an evaluation of how variations in these dynamic factors can influence the risk for violence over time and how emerging profiles may be more associated with such behavior. More specifically, there is evidence to suggest that positive psychotic symptoms are significantly related to violence, but no studies have investigated the fluctuation of these symptoms over time and the relationship between these variations and violence/criminality (9).Furthermore, anger and hostility have been shown to be associated with violence among psychiatric patients and have been associated with impulsivity, resulting in a heightened likelihood of aggressive behavior (11). However, in comparison to other negative emotional components such as anxiety and depression, less is known about the construct of anger and how its patterns in time influence violence outcomes, which warrants more studies. Moreover, beyond symptomatology, a wealth of research has shown that substance use and substance use disorder (SUD) are among the most crucial dynamic risk factors established in individuals with mental disorders (3, 4, 12, 13). Alcohol has been the substance most frequently studied and cited as being related to subsequent aggressive and violent behavior (14). Fewer studies have examined the relation between the use of illicit drugs, such as cocaine and cannabis, and violence, and these have yielded more ambiguous findings (15, 16). Moreover, longitudinal patterns of substance use identified in previous research were considerably heterogeneous (17). All in all, there have been a limited number of high-quality longitudinal studies that investigated the relationship between particular psychiatric symptoms, types of substances, and specific criminal and/or violent outcomes (18). Also, studies have had important shortcomings. For example, they have focused on a limited set of drugs (e.g., alcohol only, alcohol and cocaine only) or have combined different types of drugs, making it difficult to assess the potential unique effects of other drugs. Other limitations include focusing exclusively on history of violence or overall violence, and not distinguishing among different types of violent acts or longitudinal profiles of violent offenders.

This longitudinal study thus aims to extend prior research by taking into account the dynamism of important risk factors of violence and criminality such as psychotic and mood symptomatology and substance use patterns while considering other confounding factors to better predict violence and/or criminality. In an attempt to more fully describe the longitudinal patterns of problematic behavior, growth mixture trajectory analytical techniques will be employed to categorize individuals based upon common attributes (e.g., levels of violence/crime, substance use, and symptomatology over time). This will likely aid in understanding how certain profiles interact and are associated with patterns of violence and/or criminality. We hypothesized that trajectories with higher levels of polysubstance use (cannabis, cocaine, and alcohol) and negative affect problems (i.e., hostility, anger) in recently discharged psychiatric patients from the *MacArthur Violence Risk Assessment Study* (MVRAS) will be strong predictors of higher violence as well as criminality trajectories, while accounting for a variety of confounding factors of violence.

## Methods

### Study Design

The participants were part of the MVRAS, which comprised 1,136 male and female patients who were recruited before their discharge from three different psychiatric facilities chosen for their geographic and patient diversity (Western Missouri Mental Health Center, Kansas City; Western Psychiatric Institute and Clinic, Pittsburgh; Worcester State Hospital and the University of Massachusetts Medical Center, Worcester). Inclusion criteria included patients who were aged between 18 and 40; spoke English; were Caucasian or of African-American ethnicity (or Hispanic, in Worcester only); and had a chart admission diagnosis of a psychotic, mood, substance, or personality disorder. Subjects were excluded if they had been hospitalized for over 21 days before being admitted.

Data were collected between 1992 and 1995; patients completed a baseline interview in the hospital and five subsequent interviews every 10 weeks during their first year following their discharge. Interviews with the patients were conducted in person (89%) or by telephone (11%). During each interview, the patient had to choose a collateral (i.e., a person who was the most familiar with their behavior in the community), or the interviewer suggested the most appropriate person based on a review of the subject’s social network data. Collateral interviews were then conducted (45% in person and 55% by telephone). Finally, records on arrests obtained at the end of the 1-year follow-up period were also a source of information for the patient’s behavior in the community. These records contained arrests that occurred during each follow-up period. The detailed protocol of the MVRAS can be found elsewhere (19).

During the study period, 12,873 persons were admitted, of whom 7,740 met the eligibility criteria, and 1,695 were approached for consent. The refusal rate was 29% (*N* = 492), and 67 enrolled subjects were released before the first interview could take place. A total of 1,136 participants were then assessed at baseline. The included participants were significantly younger, less likely to suffer from schizophrenia, and more likely to abuse drugs/alcohol and to have a personality disorder than patients who refused to participate. In our study, 825 participants were selected due to their longitudinal profile, as they had at least two follow-up assessments. Compared to enrolled patients lost to follow-up, the included participants were more likely to have a history of alcohol/drug abuse and less likely to have a chart history of violence toward others.

### Assessments

*Violence*: At each time point interview, the participants were asked several questions regarding the violent behaviors they committed during the 10 previous weeks. Violence was evaluated with the *MacArthur Community Violence Instrument* and was classified into three categories, according to the two constructs of violence labeled in published reports from the MVRAS (19, 20): 1) no violence, 2) other aggressive acts (a battery that did not result in physical injury), and 3) serious violence (a battery that resulted in physical injury, sexual assaults, assaultive acts that involved the use of a weapon, or threats made with a weapon).

*Criminality*: The level of criminality during the 1-year follow-up period was estimated with the number of arrests in the 10 weeks before each time point. This was retrieved with criminal records obtained at the end of the entire follow-up period (19). Arrests linked to substance use (e.g., selling, possession) were differentiated to assess the influence of specific substance use on other types of arrests. Crimes before baseline either against a person or against property were also considered as two dichotomous variables (i.e., presence or absence) to predict violence and criminality during the 1-year follow-up period.

*Substance use (dynamic)*: At each time point, participants were asked about their use of substances (alcohol, cannabis, and cocaine) since the previous time point. This was obtained by self-reported measures comprising the number of days in a typical week of having used alcohol, cannabis, and/or cocaine (19).

*Symptomatology (dynamic)*: Common psychiatric symptoms were assessed at each time point with the *Brief Psychiatric Rating Scale* (BPRS) (21), which is a widely used semistructured interview rated by clinicians comprising 18 items. The total score of the BPRS showed very good reliability, as expressed by the intraclass correlation (*R* = 0.78, *p* < 0.001), and a good validity, with the global estimate showing a correlation of *R* = 0.66 (*p* < 0.01) (22). Items were separated into five subscales based on Shafer (23): 1) positive symptoms (thought content, conceptual disorganization, hallucinatory behavior, grandiosity), 2) negative symptoms (blunted affect, emotional withdrawal, motor retardation), 3) affective symptoms (anxiety, guilt, depression, somatic), 4) resistance (hostility, uncooperativeness, suspiciousness), and 5) activation (excitement, tension, mannerisms–posturing).

*Psychopathic traits (static)*: The *Psychopathy Checklist: Screening Version* (PCL:SV) (24), containing 12 items, was used to assess psychopathic traits at baseline. Each item is rated on a three-point scale (0—nonapplicable; 1—possibly or partially present; 2—present). The total score ranges from 0 to 24. This tool has good psychometric properties in psychiatric patients. The PCL:SV shows good structural reliability, with a mean Cronbach’s alpha of 0.80. The total score also has good interrater reliability, with the intraclass correlation coefficient averaging around 0.80. The PCL:SV also has a good concurrent validity with the Psychopathy Checklist-Revised (PCL-R): first, its items are strongly related to the PCL-R items from which they are derived, and second, the correlation between total scores on the two tests is about 0.90 (24, 25). The baseline total score was used as a continuous variable to predict violence and criminality.

*Impulsivity (static)*: The *Barrat Impulsiveness Scale* (BIS-11) (26), a 30-item self-report questionnaire, was used to assess attentional, motor, and cognitive/nonplanning impulsiveness. For most subjects, it was administered twice, at follow-up 1 and again at follow-up 3. Each item is rated on a four-point scale (1—never/rarely; 2—occasionally; 3—often; 4—almost always/always). The total score is internally consistent and is useful to measure impulsiveness among patient and inmate populations, with a Cronbach’s alpha of 0.83 in general psychiatric patients (26). The total score (mean of the two measures) was used as a continuous variable to predict violence and criminality.

*Anger (static)*: The *Novaco Anger Scale* (NAS) (27) is a 60-item self-report questionnaire designed to evaluate anger as a problem of psychological functioning and physical health and to assess therapeutic change. For most subjects, it was administered twice, at follow-up 1 and again at follow-up 3. The NAS yields four subscale scores (cognitive, arousal, behavioral, and anger regulation) and a total score. This instrument was developed for use in both the general population and clinical samples; it can therefore be used in those with a psychiatric illness. Each item is rated on a three-point scale (1—never true; 2—sometimes true; 3—always true). Internal reliability for the total score was 0.94 (27), and the 1-month test–retest reliability ranged from 0.78 to 0.91 (28). We used the total score (mean of the two measures) as a continuous variable to predict violence and criminality.

*Imagined violence (static)*: The *Schedule of Imagined Violence* (SIV) (29) is a structured set of eight questions developed specifically for the MVRAS. Based on the answers of the participants to the first two questions at each time point, they were then assigned into two categories: 1) SIV+ or 2) SIV−. SIV+ status required them to further report ever having thoughts about physically harming others (Q1), and if so, the last time this happened had to be within the past 2 months (Q2). SIV status (+ or −, based on time points 1 to 5) was used as a dichotomous variable in predictive models for violence and criminality.

*Delusions (static)*: To assess if the participant had delusions, a set of questions was administered at each time point, which was mostly obtained from the *Diagnostic Interview Schedule* (DIS). Interviewers were then asked to judge, using the Diagnostic and Statistical Manual of Mental Disorders, 3^rd^ version, revised (DSM-III-R) definition of delusions, on the basis of all information available to them, whether the subjects were possibly/definitely delusional or whether the responses reflected reality or some other nondelusional perception. To ensure consistency, a psychiatrist reviewed all screening forms, which contained the subjects’ verbatim descriptions of their beliefs. If necessary, they were also able to listen to the audiotapes of the interview (19). The presence or absence of delusions (at follow-ups 1 to 5) was used as a dichotomous variable in predictive models for violence and criminality.

*Verbal IQ (static)*: Verbal IQ was assessed at baseline using the *Wechsler Adult Intelligence Scale—Revised* vocabulary subtest (30), which contains 35 items and has demonstrated excellent psychometric properties (30). The verbal IQ score was used as a continuous variable to predict violence and criminality.

*Social support (static)*: Social support was estimated by the size of the participant’s social network at baseline (31) with the semistructured interview entitled *Social Network Inventory* (19). Participants were asked to name every person in their social network; the number of people identified was used as a continuous variable to predict violence and criminality.

*Education level (static)*: The highest level of schooling was self-reported during the baseline interview. The variable was coded into the number of years if the participant did not complete high school, or according to the following codes: 12—high school graduate; 13—1 year of college; 14—2 years of college, 15—3 years of college; 16—college graduate; 17—some graduate schooling; 18—graduate degree; and 19/20—postgraduate studies. The number of years of education was used as a continuous variable to predict violence and criminality.

*Childhood adversity (static)*: Childhood adversity was estimated by whether participants were beaten by their parents during their childhood or adolescence and by whether their parents were beating each other. This information was self-reported in the “family history” section of the baseline interview (19). The three variables “beat as a child” (i.e., childhood victimization, up to 12 years old), “beat as a teenager” (i.e., adolescence victimization, after the age of 12), and “parents beat each other” were used as dichotomous variables (presence/absence) to predict violence and criminality.

*Previous hospitalizations (static)*: Information about previous hospitalizations (i.e., age at first hospitalization, number of hospitalizations) was self-reported during the baseline interview (19). The number of prior hospitalizations, the age at first hospitalization, and the number of years since the first hospitalization (at baseline) were used as continuous variables to predict violence and criminality.

### Statistical Analysis

*Trajectories*: Trajectories were estimated for violent behaviors, number of arrests, substance use (cocaine, cannabis, alcohol), and symptoms (positive, negative, affect, resistance, activation) throughout the follow-up using growth mixture models. The optimal number of clusters was determined by using the sample-size-adjusted Bayesian information criterion (aBIC) that showed the lowest value or the first value before only a very small decrease of aBIC was observed. Other properties of the model like entropy were also examined to ensure that the groups had good separation. A probit link was used for the violence outcome, because the variable was a three-group gradient of violence (1—no violence; 2—other aggressive acts; 3—violence). Criminality was modeled using a log link and assuming a negative binomial count distribution. Symptom scores were analyzed assuming a normal distribution. Membership indicators of subgroups of clusters were used afterward as dichotomous variables describing patterns of violence and criminality.

*Descriptive analysis*: The primary outcomes (violence and criminality) were compared against all covariates (*static risk factors*—demographics, psychopathic traits, impulsivity, anger, verbal IQ, social support, education level, childhood adversity, previous hospitalizations; *dynamic co-occurring factors*—membership in substance use subgroups, membership in symptom trajectories, violence fantasies, delusions). These variables were selected based on theoretical assumptions about the predictors of violence and/or criminality. For scales administered twice (impulsivity and anger), we used the mean scores as predictive “static” variables, as they almost did not vary across time. Pearson’s chi-squared test was used to assess dichotomous outcomes differences. For continuous variables, normality was first assessed with the Shapiro–Wilk test. Differences between them were then assessed with *t*-tests (for normal variables) or Mann–Whitney tests (for non-normal variables) reporting *p*-values.

*Logistic regressions*: Binary logistic regression models were built at the individual level, accounting for data clustering to assess associations between potential predictors and violence clusters (no/low violence versus moderate/high violence) and between potential predictors and criminality clusters (no/low criminality versus moderate/high criminality). Variables in the descriptive statistics significant at *p* < 0.1 were entered. Backward elimination of variables was then conducted, which is less likely to encounter type II errors than forward elimination (32). Removal testing was based on the likelihood-ratio test on the maximum partial likelihood estimates. At each step, the model was reestimated until all variables were significant (*p* < 0.05).

*Receiver operator characteristic (ROC) curves*: The predictive accuracy of risk assessment scores was then examined using *receiver operator characteristic* (ROC) curves for violence and criminality, separately. Graphically, the ROC measurement is often represented in the form of a curve that describes the rate of true positives (sensitivity: the fraction of positives that are actually detected) as a function of the rate of false positives (specificity: the fraction of negatives that are incorrectly detected) for each possible score on the scale. The area under the curve (AUC) represents an indicator of the overall predictive accuracy of the instrument’s scores, which allows comparison with other risk assessment tools. AUCs reflect the probability that the risk score of a randomly chosen offender would be higher than the risk score of a randomly chosen nonoffender.

*Software*: All analyses were performed using SPSS 25 (33) except for growth curve mixture models, which were estimated using Mplus Version 8 (34).

## Results

### Sample Characteristics

In the final sample of 825 subjects with a longitudinal profile, the mean age was 30.0 years (SD = 6.2; range: 18–40). The highest education level was a high school diploma (41.9%). The majority were men (57.3%), were Caucasian (66.9%), had never been married (57.8%), and had a primary diagnosis of depression (56.0%). Other diagnoses included schizophrenia (15.2%), bipolar disorder (14.2%), mania (8.7%), and schizoaffective disorder (6.2%). These primary diagnoses did not significantly differ between the violence groups or criminality groups. Furthermore, close to half the sample had had lifetime drug (49.5%) or alcohol (49.7%) dependence; 32.9% were suffering from alcohol dependence at baseline; and a quarter, from drug dependence. After the baseline, 33.4% consumed drugs at the first follow-up, 34.29% at the second, 30.6% at the third, 29.9% at the fourth, and 28.3% at the final follow-up.

Regarding violence at postdischarge, most participants showed no signs of violence or committed very few incidents (*N* = 495; 60%), while 40% (*N* = 330) were classified in the high-violence group. As for non-substance-related criminality, 103 (12.7%) fell into the group with many arrests (two to four) during the follow-up year.

### Growth Mixture Models

**Figure 1:**
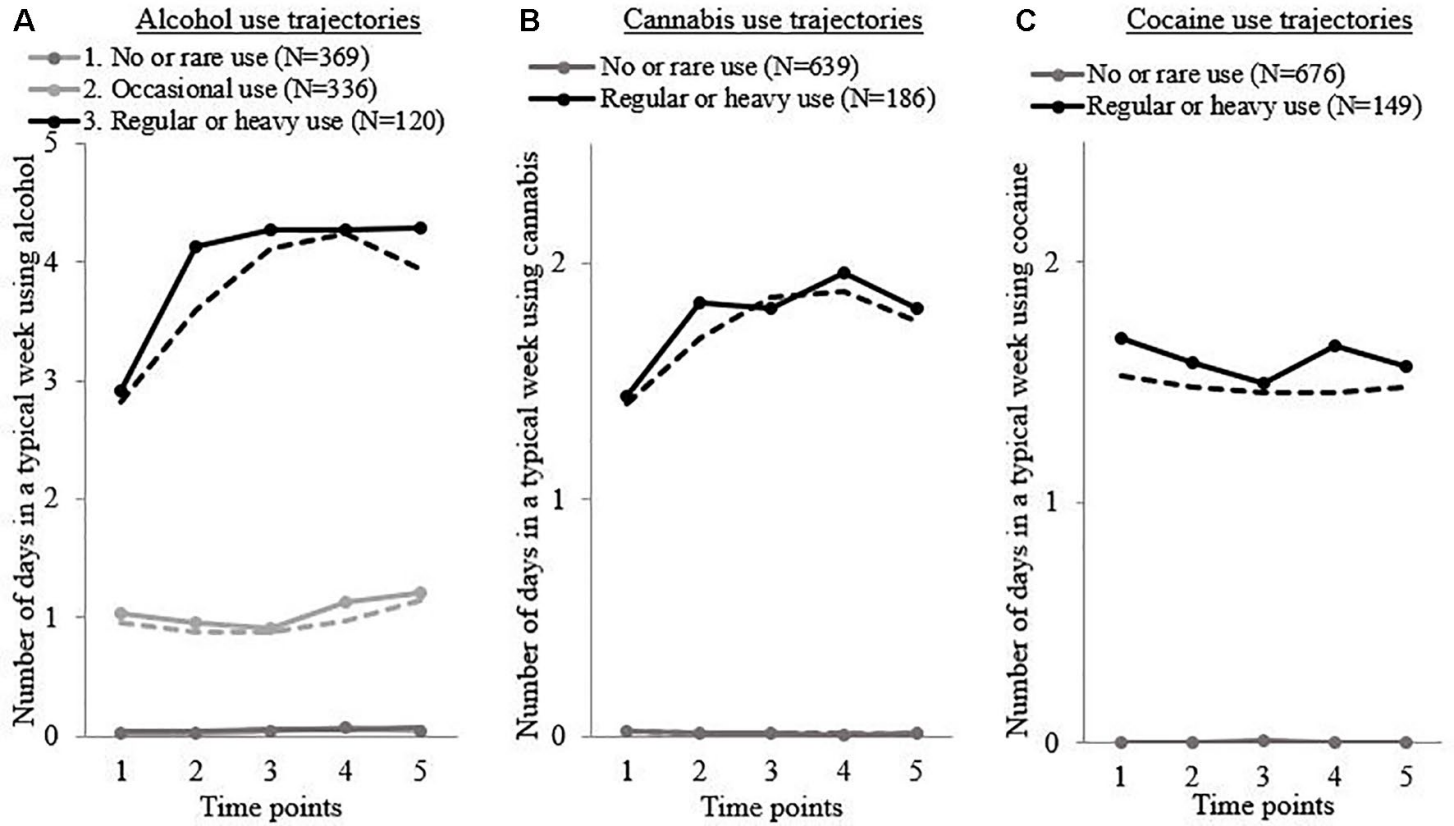
Substance use clusters based on the number of substance-using days in a typical week in recently discharged psychiatric patients across follow-up visits 1 to 5. **(A)** Alcohol use trajectories, **(B)** cannabis use trajectories, and **(C)** cocaine use trajectories. Solid lines: self-reported typical number of days using this substance, by people who probably belong to this group (probability > 0.5). Dotted lines: predicted number of days using this substance when belonging to this group, according to the model. *N* = 825.

**Figure 2:**
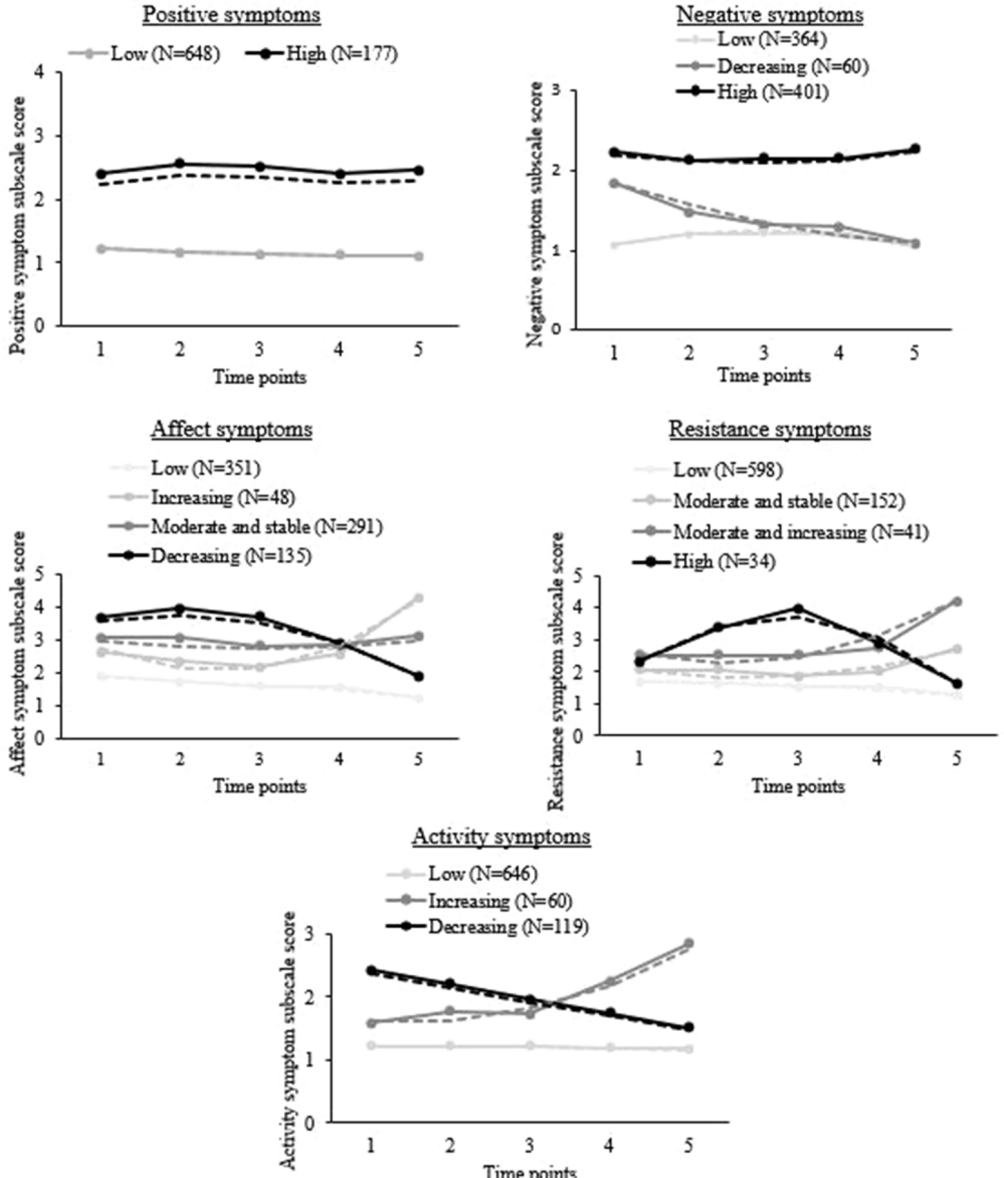
Symptom clusters based on the subscales of the Brief Psychiatric Rating Scale (BPRS) in recently discharged psychiatric patients across follow-up visits 1 to 5. Solid lines: observed symptomatology in people who probably belong to the group (probability > 0.5), as measured with the BPRS. Dotted lines: predicted symptomatology when belonging to this group, according to the model. N = 825.

Trajectories were calculated for substance use, symptoms, violence, and criminality across all five follow-ups. For most participants, membership in each trajectory was high (>0.80); these probabilities are described in the **Supplementary Material**. Substance use and symptom clusters are graphically presented in **Figures 1** and **2**, respectively.

*Substance use*: Different clusters have been calculated for alcohol, cocaine, and cannabis use, based on the number of days the subject used the substance in a typical week (see **Figure 1**). Cocaine and cannabis trajectories show two distinct profiles of individuals: the first trajectory represents individuals who never or almost never consumed, and the second, those who consumed occasionally or frequently. For alcohol, three groups were estimated: the first one shows very low (no or rare) alcohol consumption, the second represents occasional drinkers (who drink approximately 1 day per week), and the third group are considered moderate to frequent drinkers (drinking 3–4 days per week). For every group of substance users, consumption remained fairly stable over time.

The first two groups of alcohol users were merged, as they were associated with neither violence nor criminality, in order to obtain more people in each group. Afterward, trajectories were crossed to distinguish every type of substance user (those who did not consume anything versus those who consumed cannabis, cocaine, and alcohol combined), resulting in eight subgroups of substance users (see **Table 1**).

**Table 1:** Cannabis, cocaine, and alcohol user subgroups based on previously calculated substance use trajectories. *N* = 825.

	Cannabis: rare/no use (first trajectory)		Cannabis: regular/heavy use (second trajectory)	
	**Cocaine:** rare/no use (first trajectory)	**Cocaine:** regular/heavy use (second trajectory)	**Cocaine:** rare/no use (first trajectory)	**Cocaine:** regular/heavy use (second trajectory)
**Alcohol:** no use/rare/occasional (first and second trajectories, merged)	1. “No/low substance use” subgroup (*N* = 389)	2. “Cocaine only” subgroup (*N* = 37)	3. “Cannabis only” subgroup (*N* = 18)	4. “Cannabis + cocaine” subgroup (*N* = 14)
**Alcohol:** regular/heavy use (third trajectory)	5. “Alcohol only” subgroup (*N* = 153)	6. “Cocaine + alcohol” subgroup (*N* = 38)	7. “Cannabis + alcohol” subgroup (*N* = 103)	8. “Cannabis + cocaine + alcohol” subgroup (*N* = 57)

*Symptoms*: For psychiatric symptomatology, trajectories were calculated for each of the five subscales of the BPRS [(A) positive, (B) negative, (C) affect, (D) resistance, and (E) activity]. Each symptom trajectory was named after the pattern that was observed as seen in **Figure 2**.

**Figure 3:**
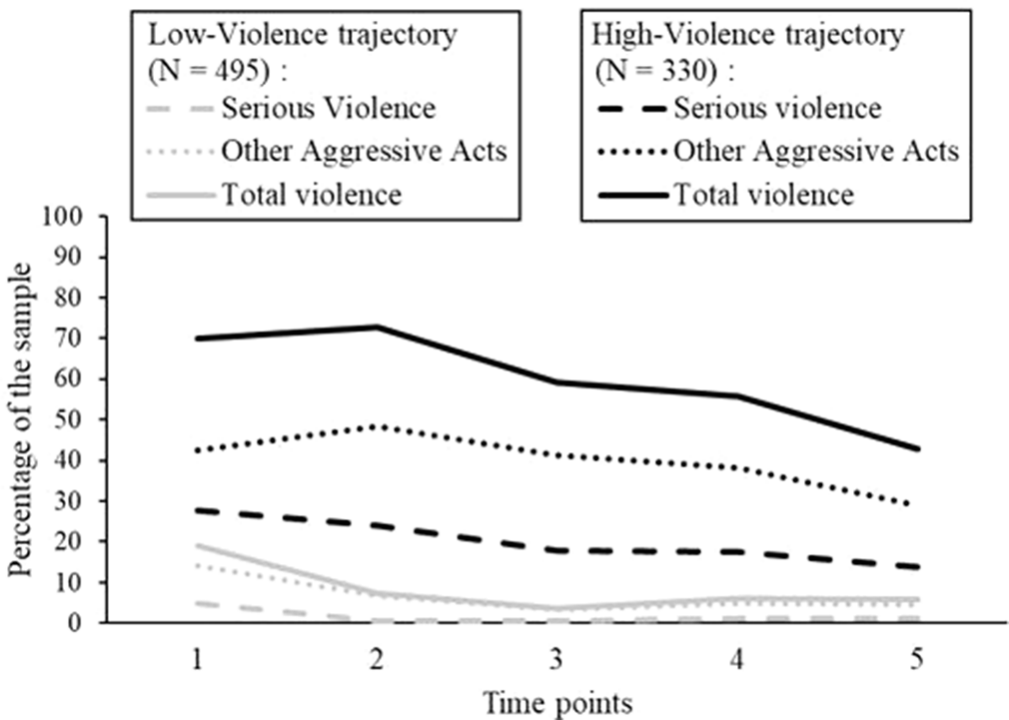
Proportion of recently discharged psychiatric patients classified into the categories of serious violence and other aggressive acts in both violence trajectories across follow-up visits 1 to 5. N = 825.

*Violence*: Trajectories for violence were built based on self-reported type of violent behaviors across the 1-year follow-up period. A two-cluster model was optimal to describe the evolution of violence following discharge. The first group expressed very few violent acts (low violence), while the second group demonstrated repeated violent and other aggressive acts (high violence) throughout the follow-up, as illustrated in **Figure 3**. Both trajectories remained quite stable across time.

**Figure 4:**
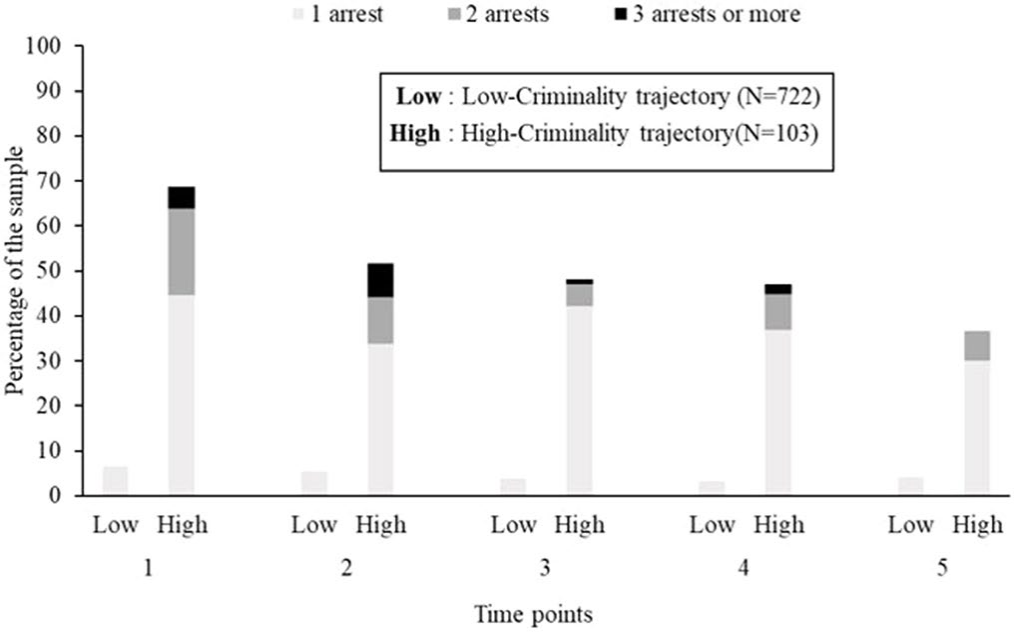
Percentage of recently discharged psychiatric patients who have been arrested and number of arrests in both criminality trajectories across follow-up visits 1 to 5. Substance-use-related arrests have been excluded. N = 825.

*Criminality*: For criminality, a two-trajectory model was optimal to describe the evolution of the number of arrests throughout the follow-up: the first group showed very few arrests (low criminality), and the second, repeated multiple arrests across time points (high criminality). Arrests in these two groups are presented in **Figure 4**. Trajectories did not increase or decrease much in time.

### Determining Variables Associated With Membership in High-Violence or High-Criminality Clusters

As described, descriptive analyses were used to identify potential predictors of violence and criminality. All variables considered in this step (including membership to substance use and symptom trajectories) are presented in **Table 2**. All variables positively associated (*p* < 0.01) with belonging (probability > 0.5) in the high-violence or high-criminality trajectories were then included in logistic regressions. Chart admission diagnoses were excluded, as they were not confirmed within the study, and the same information was more precisely covered with symptomatology and substance use. 

**Table 2:** Variables associated with higher probability of membership in high-violence and high-criminality trajectories, *N* = 825.

		Low-violence trajectory (*N* = 495)	High-violence trajectory (*N* = 330)		Low-criminality trajectory (*N* = 722)	High-criminality trajectory (*N* = 103)	
Variables	Test	Mean (SD)	Mean (SD)	*p*-value	Mean (SD)	Mean (SD)	*p*-value
Demographics (baseline)							
Age	M-W	**30.5 (6.2)**	**29.4 (6.1)**	0.010*	**30.2 (6.3)**	**29.1 (5.5)**	**0.07**
Number of people in social network	M-W	10.3 (4.9)	10.8 (5.3)	0.28	**10.7 (5.1)**	**9.2 (4.6)**	0.008*
Verbal IQ	M-W	**36.8 (16.7)**	**30.2 (15.0)**	<0.001**	**35.0 (16.6)**	**28.0 (13.7)**	<0.001**
Education level	M-W	**12.5 (2.2)**	**11.6 (2.0)**	<0.001**	**12.2 (2.1)**	**11.1 (2.0)**	<0.001**
Years since first hospitalization	M-W	8.5 (7.0)	7.7 (6.5)	0.26	8.1 (6.7)	8.7 (7.1)	0.47
Age at first hospitalization	M-W	22.4 (6.9)	21.5 (7.0)	0.18	22.2 (6.9)	20.8 (6.8)	0.22
Number of hospitalizations	M-W	6.8 (13.2)	5.4 (9.7)	0.24	**5.8 (10.9)**	**9.17 (17.5)**	**0.07**
Total scores from scales							
PCL:SV (baseline)	M-W	**6.8 (5.0)**	**11.1 (5.3)**	<0.001**	**8.0 (5.4)**	**12.6 (5.0)**	<0.001**
NAS (mean from time points 1–5)	*t*-test	**86.0 (14.4)**	**97.1 (14.1)**	<0.001**	**89.8 (15.2)**	**94.7 (14.5)**	0.004*
BIS (mean from time points 1–5)	*t*-test	**54.0 (14.1)**	**61.5 (14.4)**	<0.001**	**56.3 (14.7)**	**61.9 (14.1)**	0.001*
		% of subjects	% of subjects		% of subjects	% of subjects	
Demographics (baseline)							
Sex (male)	*X* ^2^	58.6	55.5	0.37	**55.0**	**73.8**	<0.001**
Ever married (yes)	*X* ^2^	40.0	45.2	0.15	42.0	42.7	0.89
Principal admission chart diagnosis							
Psychotic disorder	*X* ^2^	10.3	7.6	0.185	10.0	3.9	0.046*
Mood disorder	*X* ^2^	62.4	55.5	0.046*	61.2	48.5	0.014*
Substance use disorder	*X* ^2^	17.2	29.4	<0.001**	20.2	35.0	0.001**
Personality disorder	Fisher	1.8	3.0	0.343	2.6	0.0	0.153
Membership (probability > 0.5) to substance use subgroups							
1. No/rare use	*X* ^2^	57.6	31.8	<0.001**	51.0	21.4	<0.001**
2. Alcohol only	*X* ^2^	18.0	19.4	0.61	19.3	13.6	0.17
3. Cocaine only	*X* ^2^	**3.2**	**6.1**	**0.05**	**3.5**	**10.7**	0.001**
4. Cannabis only	*X* ^2^	**1.6**	**5.5**	0.002*	**2.5**	**7.8**	0.004*
5. Cocaine + cannabis	*X* ^2^	**2.2**	**3.3**	0.049*	**2.2**	**5.8**	0.033*
6. Cocaine + alcohol	*X* ^2^	**3.4**	**6.4**	0.020*	**4.0**	**8.7**	0.032*
7. Cannabis + alcohol	*X* ^2^	**10.3**	**15.8**	<0.001**	12.2	14.6	0.50
8. Polysubstance (cocaine + cannabis + alcohol)	*X* ^2^	**3.6**	**11.8**	<0.001**	**5.4**	**17.5**	<0.001**
Membership (probability > 0.5) to symptom trajectories							
Positive							
1. Low	*X* ^2^	80.8	75.2	0.05	78.5	78.6	0.98
2. High	*X* ^2^	**19.2**	**24.8**	**0.05**	21.5	21.4	0.98
Negative							
1. Low	*X* ^2^	44.2	43.9	0.93	42.9	52.4	0.07
2. Decreasing	*X* ^2^	7.9	6.4	0.41	7.3	6.8	0.84
3. High	*X* ^2^	47.9	49.7	0.61	**49.7**	**40.8**	**0.09**
Affect							
1. Low	*X* ^2^	46.5	36.7	0.005*	43.1	38.8	0.47
2. Increasing	*X* ^2^	5.9	5.8	0.95	5.7	6.8	0.65
3. Moderate and stable	*X* ^2^	**32.7**	**39.1**	**0.06**	34.8	38.8	0.42
4. Decreasing	*X* ^2^	14.9	18.5	0.18	16.5	15.5	0.81
Resistance							
1. Low	*X* ^2^	78.2	63.9	<0.001**	73.4	66.0	0.12
2. Moderate and stable	*X* ^2^	**15.8**	**22.4**	0.016*	17.9	22.3	0.27
3. Moderate and increasing	*X* ^2^	**3.0**	**7.9**	0.002*	4.8	5.8	0.67
4. High	*X* ^2^	3.0	5.8	0.05	3.9	5.8	0.35
Activation							
1. Low	*X* ^2^	82.2	78.4	0.001**	79.8	68.0	0.006*
2. Increasing	*X* ^2^	**5.7**	**9.7**	0.029*	**6.6**	**11.7**	**0.07**
3. Decreasing	*X* ^2^	**12.1**	**17.9**	0.021*	**13.6**	**20.4**	**0.07**
Other variables							
Childhood victimization	*X* ^2^	**50.9**	**57.3**	**0.07**	54.2	48.5	0.25
Adolescence victimization	*X* ^2^	38.4	41.8	0.33	40.0	37.9	0.69
Parents hit each other	*X* ^2^	32.3	36.4	0.17	33.2	38.8	0.39
Prior arrests for crimes against property (before baseline)	*X* ^2^	**31.9**	**46.1**	<0.001**	**36.0**	**48.5**	0.014*
Prior arrests for crimes against person (before baseline)	*X* ^2^	**18.0**	**30.3**	<0.001**	**21.1**	**35.9**	0.001**
Schedule of Imagined Violence (time points 1–5)	*X* ^2^	**24.8**	**39.1**	<0.001**	30.5	31.1	0.92
Presence of delusions (time points 1–5)	*X* ^2^	**31.5**	**24.2**	0.024*	29.4	23.3	0.20

M-W, Mann–Whitney test; X^2^, chi-square test; PCL:SV, Psychopathy Checklist: Screening Version; NAS, Novaco Anger Scale; BIS, Barrat Impulsiveness Scale; BPRS, Brief Psychiatric Rating Scale.

*p ≤ 0.05; **p ≤ 0.001; **bold**: included in logistic regressions (next step).

### Binary Logistical Regression Models and Receiver Operator Characteristic Curves

After conducting a backward elimination of these variables in binary logistical regression, five-factor models emerged for the prediction of both violence and criminality. The model for violence at postdischarge is presented in **Table 3**. Due to missing data, 690 participants were included in the final model (83.6% of the total sample). Predictive factors for violence included regular use of cannabis (OR = 3.098), affect symptoms that remained constant over time (OR = 1.473), psychopathic traits evaluated with the PCL (OR = 1.130), anger as measured with the NAS scale (OR = 1.040), and verbal IQ (OR = 0.988). For every one-unit increase in continuous variables such as the PCL and NAS scores, the odds of being in the violent group increased by 1.129 and 1.039, respectively. For the verbal IQ score, for each one-unit decrease, the odds of violence increased by 1.013. This final model predicted 69.6% of violence and had an AUC of 0.773 (95% CI = 0.738–0.808; *p* < 0.001).

**Table 3:** Logistic regression model for predicting membership (probability > 0.5) to the high-violence trajectory, *N* = 690.

Predictive factors	S.E.	OR	95% CI	*p*-value
Membership in the “cannabis only” subgroup	0.511	3.098	1.139–8.427	0.027
Membership in the “moderate and stable” affective symptom trajectory	0.184	1.473	1.027–2.112	0.035
PCL:SV total score	0.018	1.130	1.091–1.170	<0.001
NAS total score	0.006	1.040	1.027–1.053	<0.001
Verbal IQ	0.006	0.988	0.977–0.999	0.033

S.E., standard error; OR, odds ratios; CI, confidence interval; PCL:SV, Psychopathy Checklist:Screening Version; NAS, Novaco Anger Scale.

Regarding criminality, the model is presented in **Table 4**. Due to missing data, 681 participants were included in the final predictive model (82.5% of the sample). Substance use subgroups predictive of criminality were the “cannabis only” subgroup as well as the “polysubstance use” subgroup (cocaine + cannabis + alcohol). This model also retained the PCL score and years of education as predictive factors. As opposed to the violence model, male sex was also a determinant. The final criminality model predicted 88.5% of criminality and had an AUC of 0.788 (95% CI = 0.743–0.832; *p* < 0.001).

**Table 4:** Logistic regression model for predicting membership (probability > 0.5) in the high-criminality trajectory, *N* = 681.

Predictive factors	S.E.	OR	95% CI	*p*-value
Membership in the “cannabis only” subgroup	0.532	3.744	1.321–10.612	0.013
Membership in the “cocaine + cannabis + alcohol” subgroup	0.376	3.772	1.804–7.886	<0.001
Male sex	0.286	1.799	1.027–3.153	0.040
PCL:SV total score	0.025	1.113	1.060–1.168	<0.001
Education level	0.066	0.785	0.689–0.894	<0.001

S.E., standard error; OR, odds ratios; CI, confidence interval; PCL:SV, Psychopathy Checklist:Screening Version.

## Discussion

This longitudinal study aimed to identify static (e.g., history of violence) and dynamic factors (substance use and symptomatology) associated with violent and criminal behavior in a group of patients having recently been discharged from a psychiatric facility. Growth-based modeling allowed the identification of the different profiles of patients with varying levels of symptomatology and substance use across a 1-year period. This method is thus innovative, and its application is relevant as it allows us to consider change over time, whether it is in the intensity of symptoms or the frequency of drug consumption. Furthermore, substance use trajectories were crossed to distinguish each type of consumer (i.e., those who consumed only one substance regularly versus those who consumed more than one). To our knowledge, this is the first study using longitudinal profiling (e.g., trajectory analyses) to predict violence and criminality in adult psychiatric patients.

### Predicting Violence

Violence correlates are well studied in populations with severe mental disorders. However, there are very few high-quality longitudinal studies that have investigated the relationship between psychiatric symptoms, types of substances, and specific violent outcomes. Conducting backward elimination on binary logistic regression models, this study identified three independent static predictors of persistent violent behavior following psychiatric discharge: low verbal intelligence, high psychopathy scores, and high anger scores. Our findings on these static predictors of violence are consistent with previous literature and include high psychopathy (25, 35, 36); low verbal IQ; drug use; low levels of education (25); and high levels of anger, impulsivity, or symptomatology of severe mental disorders (36).

As for dynamic risk factors, two of them were linked to membership in the high-violence trajectory: membership in the “cannabis only” subgroup and in the “moderate and stable” affective symptom trajectory. A study by Yang and Mulvey (37) also found affective symptoms to be a predictor of violence in patients with depression followed for a year. However, only positive and affective symptoms were evaluated. On the other hand, our study included negative, resistance, and activity subscales of the BPRS, which help to provide a wider view of the influence of symptoms on violence. Nonetheless, only the group with high and stable affective symptoms remained significant when controlling for other variables, which is consistent with previous research (37). Substance use, a well-known risk factor for violence, may also lead to repeated violence (19, 20, 38, 39). However, in our multivariate analysis, only the trajectory of cannabis use, on the contrary to alcohol and cocaine, was an independent predictor when controlling for psychopathy, anger, affective symptoms, and verbal IQ. This result is unexpected because alcohol has previously been shown to be related to violence with more evidence as compared to cannabis (40). Notably, this study has shown the independent effect of the use of cannabis alone to predict violence, which differs from prior studies. Whereas literature has found similar associations between cannabis use and violence in psychiatric populations (3, 41–45), none has used longitudinal profiling of substance use and controlled for important confounding factors. The available data in this study did not allow us to explain how cannabis use is associated with an increase in violence; nevertheless, research has shown that the effects of cannabis on violence may be increased during either intake or withdrawal (46, 47). Cannabis use could also exacerbate symptomatology and aggressivity (48–52), which may then raise the risk of violence. Future studies are necessary to illuminate this relationship.

### Predicting Criminality

Risk factors for criminal behaviors have been well documented in forensic populations; however, less attention has been paid to psychiatric patients living in the community. The model used in our study allowed the identification of predictors of membership in a high-criminality cluster with a moderate predictability, which differed slightly from the factors found for violence. 

While levels of psychopathy and membership in the “cannabis only” subgroup remained significant, “polysubstance” use (membership in the cocaine + cannabis + alcohol subgroup), education level, and male sex were independent predictors of criminality as well. These results are in accordance with previous findings among individuals with mental illnesses, which include a history of arrests, male sex (53, 54), chronic anger (55), and high psychopathy score (56–58).

Substance use has also been known to be associated with criminality and criminal recidivism, particularly the combination of alcohol and drugs (59, 60). As was revealed in a meta-analysis including 30 studies on the association between drug misuse and crime, cocaine use (effect size = 2.62, *p* = 0.0001) was associated with a higher risk of criminality than cannabis use (effect size = 1.51, *p* = 0.0001) (60). On the other hand, some studies included supply crime (61, 62), which should be noted since cocaine users may be more likely to be arrested for drug-related crimes (63). Moreover, the pharmacological effects of polysubstance use may increase impulsivity in a cumulative and interactive manner (64–66), augment the probability of engaging in risky criminal behaviors, and subsequently lead to more arrests. Criminal behavior could also be the result of the effects of withdrawing from one or more substances (67). However, based on the available literature, we were not able to explain the relationship between cannabis alone and polysubstance use, which requires further investigations.

## Limitations

This longitudinal study using growth-based modeling to predict violence and criminality in a psychiatric population by taking into account the dynamism of risk factors is clearly innovative. However, the study has limitations that are worth being acknowledged. First, as attrition is an inevitable limitation of research, some individuals had missing data and may have evolved differently over time. To minimize the impact of a possible attrition bias, only participants with a longitudinal profile (i.e., two or more assessment measures) were included. Second, it was not possible to evaluate the daily frequency and quantity of substance use since only the self-reported number of days the participant consumed a substance in a typical week was available. Future studies should therefore gather more information on substance use. Third, even though studying co-occurring phenomena is interesting and novel, this methodology makes it impossible to access the directionality of the association between dynamic variables and violence and/or criminality, since they occurred at the same time. It is also important to mention that data were collected more than 20 years ago, even though there is no reason to believe that these associations would have changed today. Nevertheless, the types of drugs used nowadays may be different. For instance, the level of Delta-9-tetrahydrocannabinol (THC) in cannabis may be higher today (68). Finally, all data used in this study were self-reported, except for arrests. Studies should attempt to replicate these results using, for instance, biological measures of substance use such as urine or hair drug screening tests.

## Conclusion

In conclusion, our findings regarding static predictors of violence are consistent with previous work and are relevant to better understanding the relationship between specific substance use and violence. Unexpectedly, belonging to the “cannabis only” subgroup was an independently linked predictor of membership in the high-violence trajectory when controlling for important factors such as psychopathy level and polysubstance use (membership in the “cocaine + cannabis + alcohol” subgroup). Anger (NAS total mean score) and affective symptoms (membership in the third trajectory, “moderate and stable”) were strongly associated with belonging in the high-violence trajectory as well. For criminality, we found that cannabis use (“cannabis only” subgroup), polysubstance use (“cocaine + cannabis + alcohol” subgroup), male sex, low educational level, and high psychopathy score were associated with belonging in the high-criminality trajectory. These results are novel because very few studies have been interested in predicting criminal behaviors/arrests in a psychiatric population. Finally, our results emphasize the need to study more rigorously the impact of longitudinal patterns of specific substance use and high affective symptoms, and to evaluate more profoundly the predictors of crime, separately from violence. Also, the identified longitudinal predictors could eventually be used to improve violence and criminality risk assessment tools specifically for general psychiatric patients about to be discharged by distinguishing among different profiles of individuals who use substances.

## Ethics Statement

The study was approved by the local ethics committee from each site’s institutional review board. After a complete description of the study, all participants provided their written informed consent.

## Author Contributions

AD and SP contributed to the conception of the study. Analysis was conducted by MB and C-EG, and the manuscript was written by MB (1/2), LD (1/4), and ML (1/4). All authors contributed to the interpretation of the data and revised the content critically, and then approved the final version.

## Author’s Note

The authors of the MacArthur Violence Risk Assessment Study have shared publicly and gratuitously their database (see: http://www.macarthur.virginia.edu/read_me_file.html).

## Funding

LD and AD are funded by grants from the FRQS (Fonds de recherche Santé Québec), and MB is funded by a scholarship from the CIHR (Canadian Institutes of Health Research).

## Conflict of Interest Statement

The authors declare that the research was conducted in the absence of any commercial or financial relationships that could be construed as a potential conflict of interest.
