# Supplementary material for: Trajectories of Dynamic Risk Factors as Predictors of Violence and Criminality in Patients Discharged From Mental Health Services: A Longitudinal Study Using Growth Mixture Modeling
Source: Front Psychiatry. 2019 May 9;10:301. doi: 10.3389/fpsyt.2019.00301 (PMC6520437; doi:10.3389/fpsyt.2019.00301)
Supplement: Supplementary file 1 [file Table_1.pdf]

Supplementary Table I. Probabilities for membership in substance use, symptoms, violence and criminality trajectories.

Trajectories	Mean probability for people who probably belong in the cluster (probability > 0.5)	Median probability for people who probably belong in the cluster (probability > 0.5)	Standard deviation
**Substance use trajectories**			
Alcohol			
No or rare use (N=369)	0.923	0.979	0.092
Occasional use (N=336)	0.884	0.939	0.127
Regular or heavy use (N=120)	0.919	0.997	0.136
Cannabis			
No or rare use (N=639)	0.976	0.996	0.065
Regular or heavy use (N=186)	0.965	1.000	0.091
Cocaine			
No or rare use (N=676)	0.976	0.987	0.052
Regular or heavy use (N=149)	0.973	1.000	0.095
**Symptom trajectories**			
Positive			
1. Low (N=648)	0.942	0.969	0.078
2. High (N=177)	0.946	1.000	0.116
Negative			
1. Low (N=364)	0.930	0.971	0.103
2. Decreasing (N=60)	0.820	0.889	0.151
3. High (N=401)	0.965	1.000	0.105
Affect			
1. Low (N=351)	0.812	0.890	0.159
2. Increasing (N=48)	0.871	0.943	0.158
3. Moderate & stable (N=291)	0.855	0.948	0.170
4. Decreasing (N=135)	0.849	0.940	0.172
Resistance			
1. Low (N=598)	0.918	0.997	0.134
2. Moderate & stable (N=152)	0.925	0.983	0.130
3. Moderate & increasing (N=41)	0.885	0.980	0.168
4. High (N=34)	0.781	0.828	0.221
Activation			
1. Low (N=646)	0.946	0.987	0.095
2. Increasing (N=60)	0.932	0.996	0.109
3. Decreasing (N=119)	0.864	0.951	0.169
**Violence trajectories**			
Low-violence (N=495)	0.894	0.958	0.119
High-violence (N=330)	0.869	0.946	0.147
**Criminality trajectories**			
Low-criminality (N=722)	0.931	0.979	0.089
High-criminality (N=103)	0.827	0.860	0.153
